# Can a multisensory teaching approach impart the necessary knowledge, skills, and confidence in final year medical students to manage epistaxis?

**DOI:** 10.1186/1916-0216-42-51

**Published:** 2013-10-09

**Authors:** George Kurien, Vincent L Biron, Chase Campbell, David WJ Cote, Kal Ansari

**Affiliations:** 1 Division of Otolaryngology-Head and Neck Surgery, University of Alberta, 1100 Youville Dr., 4016 Grey Nuns Hospital, Edmonton, AB T6L 5X8, Canada

## Abstract

**Objective:**

The purpose of this study is to evaluate the efficacy of a multisensory teaching approach in imparting the knowledge, skills, and confidence to manage epistaxis in a cohort of fourth year medical students.

**Methods:**

One hundred and thirty four fourth year medical students were recruited into the study from Aug 2011 to February 2012 in four groups. Students listened to an audio presentation (*PODcast*) about epistaxis and viewed a video presentation on the technical skills (*VODcast*). Following this, students completed a 5-minute Individual Readiness Assessment Test (IRAT) to test knowledge accrued from the *PODcast and VODcast*. Next, students observed a 10-minute expert demonstration of the technical skills on a human cadaver and spent half an hour practicing these techniques on cadaver simulators with expert guidance. The students’ confidence was assessed with Confidence Level Questionnaires (*CLQs*) before and after their laboratory session. The skill level of a subset of students was also assessed with a pre- and post-laboratory Objective Structured Assessment of Technical Skills (*OSATS*).

**Results:**

Eighty two percent of the participants achieved a score of at least 80% on the IRAT. The CLQ instrument was validated in the study. There was a statistically significant improvement between the pre- and post-laboratory *CLQ scores* (*p*<*0*.*01*) and also between pre- and post-laboratory OSATS *scores* (*p*<*0*.*01*). Qualitative feedback suggested a student preference for this teaching approach.

**Conclusions:**

This study provides further evidence that a multisensory teaching intervention effectively imparts the necessary knowledge, skill and confidence in fourth year medical students to manage epistaxis.

## Introduction

Epistaxis is a common condition that up to 60% of the population will experience. A needs-assessment survey conducted in 2000 at the University of Alberta medical school revealed that 95% of the 100 students did not have the confidence to technically manage epistaxis. This presents a significant need for effective teaching of this essential medical skill in the Canadian education curriculum.

It has been suggested that medical skills are effectively taught through multisensory approaches based on Fleming’s VARK (visual, auditory, read/write, kinesthetic) model, which proposes that some learners have a preferential sensory channel through which they best receive and integrate information [1-3]. In addition, teaching of technical skills has seen increasing use of simulation-based learning [4-6]. Utilizing cadaver simulators provides a risk free and anatomically high fidelity environment for learners to perform the technical maneuvers to control epistaxis. The majority of our learners also belong to, the “Millennial”, or Generation Y. This generation is technology savvy, resourceful, and able to multitask.

The purpose of our study was to evaluate the efficacy of a multisensory teaching approach (consisting of a *PODcast*, *VODcast*, written notes, and expert-guided practice on cadaver simulators) in imparting the necessary knowledge, skills, and confidence to technically manage epistaxis in a cohort of fourth year medical students.

## Methods

Appropriate learning objectives were created and teaching and assessment methods were matched. The course content was prepared by an otolaryngologist and was based on current literature and practice guidelines. The content validity of the teaching materials and session was ensured through a standardized checklist peer review process carried out by two other otolaryngologists. Institutional health research ethics board (University of Alberta Health Research Ethics Board) approval was obtained. A focus group of fifteen medical students ensured that the intervention was at an appropriate level of understanding.

The learning objectives were as follows:

At the end of the teaching session, the learner will be able to

1. Formulate a differential diagnosis for epistaxis and identify risk factors.

2. Prescribe appropriate medical management of epistaxis.

3. Determine when to refer the patient to an otolaryngologist.

4. Perform an examination using a nasal speculum and suction while adhering to universal precautions.

5. Perform silver nitrate cautery of the anterior nasal cavity.

6. Perform anterior nasal packing with Merocel© nasal packs.

7. Perform anterior nasal packing with Vaseline gauze.

An online Wiki hosted the learning objectives, pre-session teaching materials, and schedule. By hosting our teaching materials on the internet (Wiki, POD/VODcasts), our students were able to access teaching materials at a place and time that was convenient for them [7]. As part of the pre-session teaching materials, students listened to a 10-minute audio PODcast (iTunes and MedEdPortal) covering learning objectives 1 to 3. They also viewed a 15-minute VODcast highlighting learning objectives 4 to 7 and a 2-minute VODcast of anterior nasal packing with Vaseline gauze of on a clear plastic model [5]. Supplementary written notes were also provided covering all learning objectives. Students were informed that an IRAT would be administered at the beginning of the classroom session, ensuring they had acquired the requisite knowledge for the cadaver simulator lab.

All fourth year students participated in the epistaxis teaching session. This session was part of the otolaryngology half day offered four times per year with an average of 36 students per session. Students were informed at the start that the study would have no impact on their assessment for academic promotion and that they could withdraw from the study at any time. Participation in the teaching session was mandatory, however participation in the study was not. No personally identifiable information was gathered, and students willing to participate signed a consent form.

Students completed a 7-minute multiple-choice question IRAT assessing the knowledge they had acquired for all 7 learning objectives. Over the next 10 minutes, a facilitator discussed the answers of the IRAT with the learners while they completed a pre-cadaver session Confidence Level Questionnaire (CLQ). The CLQ assessed the student’s confidence in performing the technical learning objectives 4 through 7. The CLQ was constructed using a five point Likert scale ranging from the lowest level of confidence where the individual would not attempt the procedure, to the highest level of confidence where the individual would feel comfortable teaching it to another learner [Additional file 1: Epistaxis Questionnaire]. Each increment in the scale represented an increasing level of independent practice in the medical learner, which is more intuitive and applicable than having an arbitrary Likert scale with no attached definition. This questionnaire was reviewed with otolaryngologists at our institution for content validity, and a focus group of medical students was interviewed for understandability. Cronbach’s alpha was calculated for internal reliability. Construct validity was determined by comparing the results of the CLQ administered to thirteen experienced practitioners (Otolaryngology residents and Otolaryngologists) to thirteen randomly selected students who had not yet practiced on the cadavers.

Twenty-eight fourth year students were randomly selected prior to the cadaver session to perform the four core technical skills while being assessed by two independent observers with the Objective Structured Assessment of Technical Skill (OSATS) [Additional file 2: Epistaxis OSATS]. The fourth skill (Vaseline gauze packing) was divided into two components due to its’ increased complexity. A ‘1’ was assigned if the learner performed the skill satisfactorily at the level of a general practitioner, and a ‘0’ was assigned if this was not met. A binary scale was used to simplify the assessment for the observers and also improve the overall inter-rater reliability. A global assessment of overall performance using Likert scale of 1 through 5 was utilized at the end. This component of the instrument was adapted from Martin et al. [8]. In Doyle et al’s study [9], the instrument demonstrated excellent internal reliability (Cronbach alpha 0.91) and good validity. As this was a new instrument adapted to assess the achievement of technical skills for management of epistaxis, inter-rater reliability was determined. Two other board certified otolaryngologists verified content validity of the instrument. OSATS were performed only on a limited number of students due to limitations of student availability in an increasingly constricted curriculum and lack of trained observers.

The cadaver lab consisted of an instructor-led demonstration of the technical skills followed by practice in pairs by the students. Two to three otolaryngology residents and up to three otolaryngologists provided feedback. At the end of the session, all participants were asked to complete the post-cadaver lab CLQ and a qualitative feedback form. The previously selected twenty-eight participants had a post-cadaver OSATS administered again by the same two independent observers. We compared the pre and post-cadaver lab CLQ scores. An *a priori* sample size calculation with a Bonferroni correction was done to address the multiple comparisons being made. For a predetermined power of 0.8 and p < 0.01 (4 independent comparisons), a medium effect size (Cohen’s d=0.50), and an 80% response rate, 120 participants were required per group. Furthermore, we calculated the percentage of students that achieved a confidence level score of 3 or above on all sections at the end the session (ie: will attempt procedure with attending back up but no active involvement). This is the level of competence that is expected of residents which coincides with the next training period we are preparing our medical students for. We then compared the pre and post-cadaver lab OSATS scores. We also determined the percentage of students that achieved all 1’s and at least 3 to 5 on overall technical performance. *A priori* sample size calculation was done for the OSATS. For a predetermined power of 0.8 and p < 0.05, and a large effect size (Cohen’s d=0.80), 26 participants were required per group.

## Results

**Figure 1:**
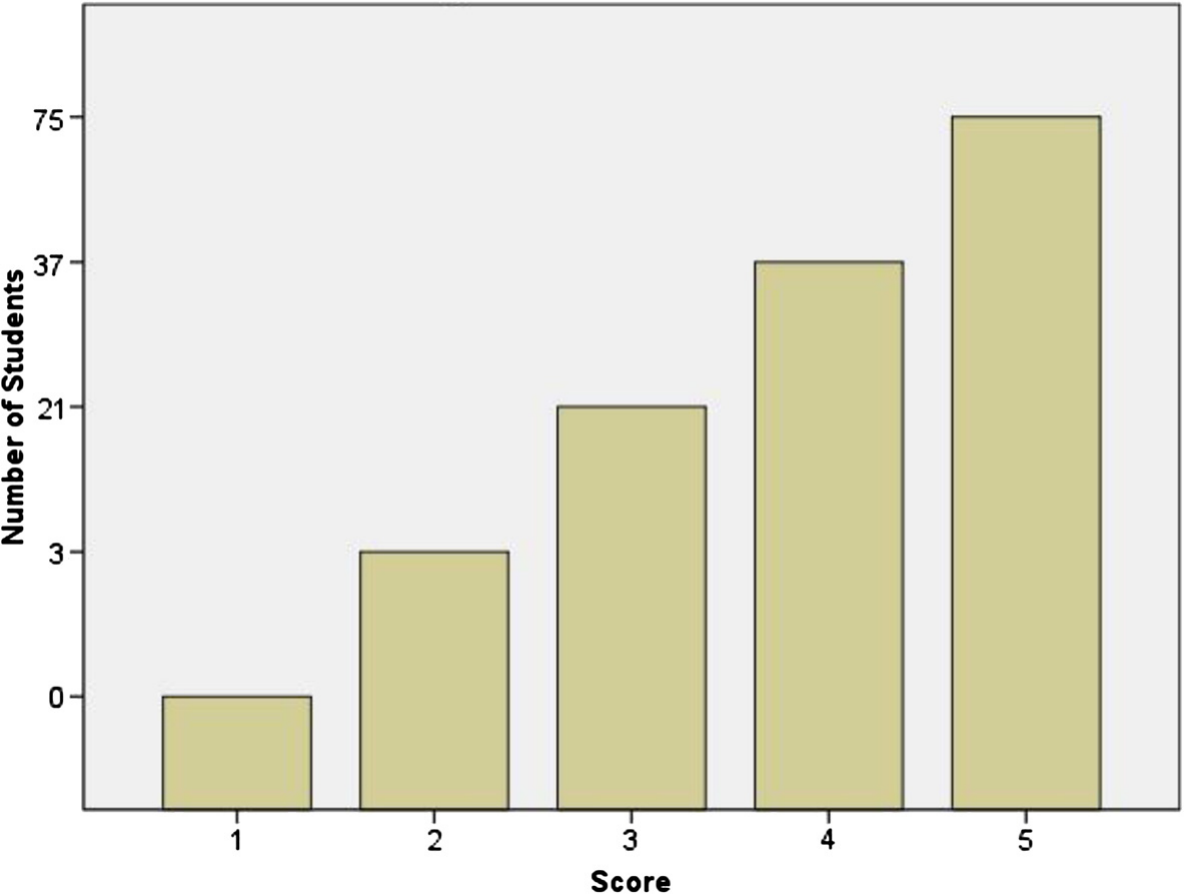
IRAT scores.

A total of 147 students participated in the teaching sessions from August 2011 to February 2012. One hundred and thirty four students provided informed consent and completed pre and post-CLQ’s, and the IRAT. Eighty-two of the 134 students received a score of 80% or higher on IRAT indicating an adequate grasp of the knowledge provided by the PODcast, VODcast, and written notes. [Figure 1 – IRAT Scores].

The internal reliability of the CLQ was calculated using Cronbach’s alpha (coefficient of reliability). Both the pre-session and post-session CLQ’s had high measures of internal reliability with alpha values of 0.85 and 0.88 respectively [SPSS 19]. Construct validity (the ability of the questionnaire to measure confidence level) was assessed by comparing the pre-session questionnaire responses of 13 randomly selected students [random.org] with the responses of a cohort of 13 otolaryngology residents and staff otolaryngologists. On all four questions, the absolute confidence scores of the experienced group were consistently higher than the pre-cadaver teaching CLQ scores for the medical students. The Mann–Whitney U test of independent samples (non-parametric) showed a statistically significant difference (p<0.01) between the groups for all four questions.

Similarly, the OSATS instrument was also validated in this study. Inter-rater reliability on each skill was calculated using Cohen’s kappa with values ranging from 0.48 to 0.85 for the pre-session OSATS to 0.65 to 1.00 for the post-session OSATS. Cronbach’s alpha was calculated for inter-rater reliability on overall performance and was found to be 0.80 pre and 0.56 post [SPSS 19].

**Figure 2:**
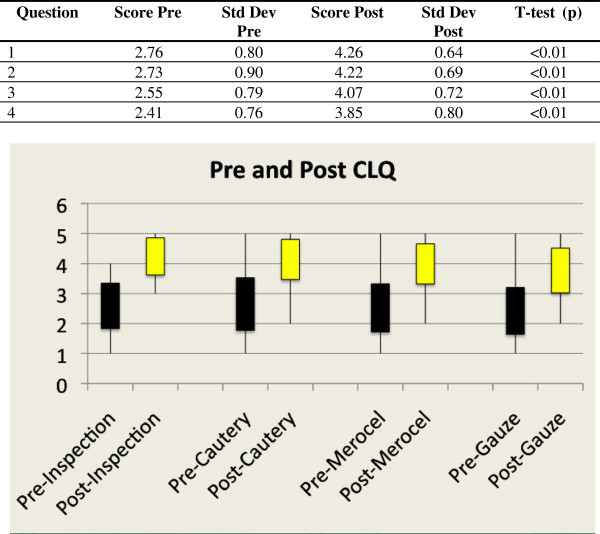
CLQ scores.

Ninety-eight percent of students achieved a score of 3 to 5 on each category (ie: will attempt procedure with attending back up but no active involvement). At baseline, students appeared to be more confident with basic procedures such as nasal cavity examination and silver nitrate cautery, and showed a decreasing trend in confidence with more complex maneuvers such as Vaseline coated gauze packing. Following the cadaver lab, students showed a clear increase in confidence for all 4 learning objectives. Using a paired two-tailed t-test (p<0.01), a statistically significant difference was found in each of the four questions between the pre and post-session responses with consistently higher scores on the post compared to the pre-cadaver teaching session [Figure 2 – CLQ Scores].

**Figure 3:**
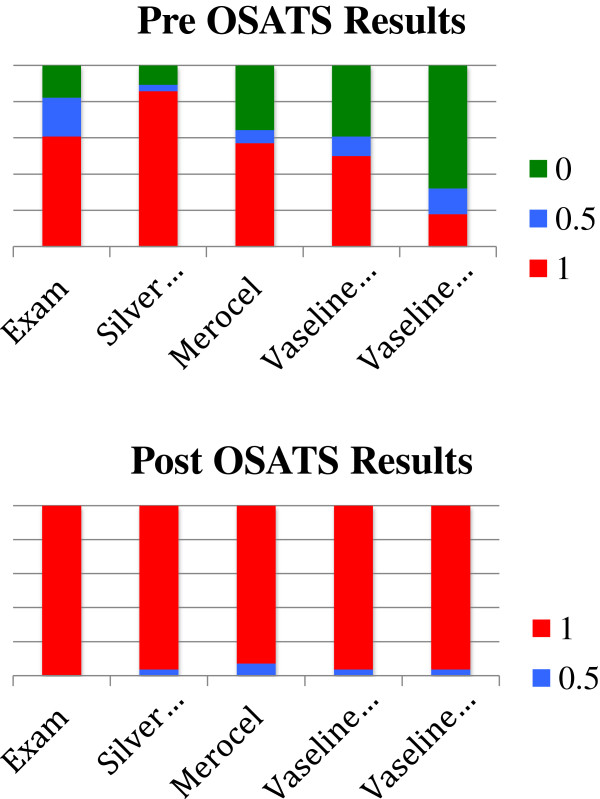
Pre and post OSAT scores.

Twenty-eight students were randomly selected to be assessed with a pre and post-teaching session OSATS. Average scores between the two observers on each of the five sections were tallied for each participant on the pre and post-OSATS [Figure 3 – Pre and Post OSATS Scores]. Like the pre-CLQ instrument, there was a trend of more students at baseline performing satisfactorily with less complex procedures than the more advanced ones. Overall scores were 2.75 (±0.67) on the pre-OSATS and 4.00 (±0.67) on the post-OSATS. On the post-OSATS, 94% of students received a score of 1 on each category and 3 to 5 on overall performance. The McNemar change test was used to compare the pre to the post-OSATS scores. A statistically significant difference was found for questions 1,2,4, and 5 (p<0.05) and no difference was found for question 3 (p=0.25).

The qualitative feedback received from the students was positive overall. Many felt that they benefited most from the cadaver simulator lab with expert feedback as well as the ability to access the pre-session media at their convenience. The most common suggestion for improvement was an improved instructor to student ratio in the cadaver simulator lab.

## Discussion

The last decade has seen a paradigm shift in medical education from the traditional ‘sage on stage’ towards a ‘learner-centered’ model. There has been an increasing adoption of self-directed problem-based learning curricula [10] and adaptation to varying learning styles. Cadaver simulators provide a risk-free learning environment where the technical training of the medical student reigns supreme. In contrast to this, in the clinical environment students often have to compete with senior trainees and physicians for these procedures. While some interventions have shown improvements in measurable learning outcomes [10], there is still considerable debate and evolution in the field. Many of the models in medical education are rooted in theories of learning styles and Fleming’s VARK model is one that is widely used [1]. This study applies this concept by providing multiple modalities of instruction (and thereby multiple opportunities) for the learner to grasp a concept and learn a skill. In the surgical educational literature, Kopta describes the acquirement of new skills as occurring in three phases – cognitive, integrative, and autonomous [11]. In the cognitive phase, the learner intellectualizes the process and plans the necessary steps. In the integrative phase, the learner initiates the appropriate motor behaviour with feedback or knowledge of the results. Finally, in the autonomous phase, motor tasks are performed smoothly with little cognitive input. The pre-session teaching materials (PODcast, VODcast, and notes) in this study aims at providing an environment for the cognitive phase while the cadaver simulators with expert guidance provide the integrative phase. This sets the stage for their continued learning in the clinical environment as they approach the autonomous phase of residency.

Given that we have measured both skill and confidence, we can apply Dreyfus’ model of skill acquisition to our study [12]. Dreyfus states that there are five levels of skill expertise: novice, advanced beginner, competent, proficient and then expert. Uniformly, most of our learners started out as novices, lacking skill and confidence. After the teaching intervention, we demonstrated that skill improved with a commensurate increase in confidence. However, we also noted that were some learners who had a disproportionately higher levels of confidence when compare to their actual skill level of achievement. According to Dreyfus, these are the advanced beginners who feel confident enough to be in independent practice but perhaps do not have the skill level to back it up. Fortunately, this is only an introductory course to prepare students for residency. Furthermore, an important competency to meet in residency is skill of self-assessment where our students will learn to align their self-assessments with objective external evaluations.

The results of the study can be better interpreted in terms of their educational significance. Simpson’s adaptation [13] of Bloom’s taxonomy of learning [14] for the psychomotor domain places a learner at a *guided response* stage where the learner is in the early stages of learning a complex skill that includes imitation and trial and error. The intended level of achievement for the participants is not mastery of the skill, but rather advancement from their current level of skill by an amount that will change their practice during their residency.

We also demonstrated the utility of internet-based resources such as PODcasting and VODcasting that can be easily used on portable media devices. By priming the students’ knowledge with the media, we were able to dedicate a proportionately greater amount of time towards active learning exercises. Although not the primary objective of this study, we were able to validate the CLQ - which looks at self-assessment of confidence in performing a technical skill.

This study does have some limitations. We did not test the students’ knowledge prior to the intervention, so we cannot be certain that there were no other sources of knowledge apart from our intervention. However, it is important to note that this is the only formal training on this topic within the medical school curriculum. We also recognize that there could be some observer bias created by the pre and post CLQs and OSATS. As students on the CLQ and observers on the OSATS both had knowledge of assessment’s timing related to course delivery, it is conceivable that both parties, in the interest of wanting to see an improvement in confidence and skill, gave better scores on the post compared to the pre-cadaver lab assessments. This observer bias for the OSATS could have perhaps been circumvented with blinding the observers. Logistically this would not have been feasible due to a constricted curriculum and lack of trained observers. Furthermore, our study did not have a matched control group to ensure that were no other confounding factors that could have partially explained our findings. We did not recruit a separate cohort of students to serve as we felt that it would not be ethical to withhold innovative teaching materials or methods from one cohort of students. A crossover design may have circumvented this problem, but again logistically this would not have been feasible due to a constricted curriculum and lack of trained observers.

In an ideal setting we would have administered the first set of CLQs and OSATS before the PODcast and VODcast, but this was prevented by time restraints. However, one can view the pre CLQ and OSATS as the level of technical achievement gained only from the PODcast, VODcast, and notes. Clearly, the students required the cadaver lab with expert guidance to achieve acceptable levels of competence. One could argue that the pre-session teaching materials were superfluous, but in reality the pre-session teaching materials primed the students for the cadaver lab. Last, given that multiple teaching methods were utilized in a synchronous fashion, it is difficult to differentiate which one had the most significant impact on the outcomes. However, the goal of our study was not to compare or ascertain which component of our teaching intervention had the most impact, but rather if a multisensory approach influenced our outcomes.

## Conclusion

Overall, our study demonstrates that our multisensory approach imparts the necessary knowledge, skill, and confidence to manage epistaxis in the lab. We would like to direct our future research efforts to look at long-term retention, if there are improvements in patient care, and if a similar model can be adapted to teaching other procedural skills [15].

## Competing interest

The authors declare that they have no competing interest.

## Authors’ contributions

GK was primarily involved in experimental design, statistical analysis and drafting of the manuscript. VB was involved in experimental design and drafting of the manuscript. CC was responsible for data collection. DC and KA were involved in experimental design, critical review and drafting of the manuscript. All authors read and approved the final manuscript.

## Supplementary Material

Additional file 1 Epistaxis Questionnaire. Click here for file

Additional file 2 Epistaxis OSATS. Click here for file
