# Supplementary material for: Can a multisensory teaching approach impart the necessary knowledge, skills, and confidence in final year medical students to manage epistaxis?
Source: J Otolaryngol Head Neck Surg. 2013 Oct 9;42(1):51. doi: 10.1186/1916-0216-42-51 (PMC3899690; doi:10.1186/1916-0216-42-51)
Supplement: Additional file 1 — Epistaxis Questionnaire. [file 1916-0216-42-51-S1.docx]

Additional file 1 – Epistaxis Questionnaire

**Epistaxis Questionnaire**

On your first real patient presenting with active epistaxis, how confident are you in performing the following skills:

| Skill |  |  |  |  |  |
| --- | --- | --- | --- | --- | --- |
| Properly examine the nasal cavity with a nasal speculum and Frasier tip suction to identify the site of bleeding | 1 | 2 | 3 | 4 | 5 |
| Perform silver nitrate cautery of the anterior nasal cavity | 1 | 2 | 3 | 4 | 5 |
| Perform anterior nasal packing with Merocel sponges | 1 | 2 | 3 | 4 | 5 |
| Perform anterior nasal packing with Vaseline gauze and Bayonet forceps | 1 | 2 | 3 | 4 | 5 |

1 = Will not attempt procedure

2 = Will attempt procedure with hands on assistance from attending

3 = Will attempt procedure with attending backup present but no active involvement

4 = Will attempt procedure independently without any attending backup

5 = Will attempt procedure while teaching it to another colleague or student
