# Supplementary material for: Can a multisensory teaching approach impart the necessary knowledge, skills, and confidence in final year medical students to manage epistaxis?
Source: J Otolaryngol Head Neck Surg. 2013 Oct 9;42(1):51. doi: 10.1186/1916-0216-42-51 (PMC3899690; doi:10.1186/1916-0216-42-51)
Supplement: Additional file 2 — Epistaxis OSATS. [file 1916-0216-42-51-S2.docx]

Additional file 2 – Epistaxis OSATS

**Epistaxis OSATS**

Performance of specific tasks

| Item | Not done/Done Incorrectly | Done correctly |
| --- | --- | --- |
| Uses speculum and suction to examine nose appropriately | 0 | 1 |
| Demonstrates appropriate technique of silver nitrate cautery | 0 | 1 |
| Inserts Merocel into nasal cavity appropriately | 0 | 1 |
| Holds Vaseline gauze appropriately with bayonet forceps | 0 | 1 |
| Packs nose with Vaseline gauze appropriately | 0 | 1 |

Overall performance

| 1 | 2 | 3 | 4 | 5 |
| --- | --- | --- | --- | --- |
| Unsatisfactory | Poor | Average | Good | Outstanding |
| Tentative and awkward movements with incorrect steps or out of order. |  | Some unnecessary movements, but able to complete the task |  | Economy of movement and has a textbook final product |
